# Functional Analysis of the Differences in the Dimensions of Two Types of Boiled and Steamed Rice Grains

**DOI:** 10.1155/2021/5546016

**Published:** 2021-07-29

**Authors:** Mirosław Krzyśko, Waldemar Wołyński, Marek Domin, Zofia Hanusz, Leszek Rydzak, Łukasz Smaga, Andrzej Wojtyła

**Affiliations:** ^1^Interfaculty Institute of Mathematics and Statistics, Calisia University-Kalisz, Poland; ^2^Faculty of Mathematics and Computer Science, Adam Mickiewicz University, Poland; ^3^Department of Biological Bases of Food and Feed Technologies, University of Life Sciences in Lublin, Poland; ^4^Department of Applied Mathematics and Computer Science, University of Life Sciences in Lublin, Poland; ^5^Health Sciences Faculty, Calisia University-Kalisz, Poland

## Abstract

The study tested how the cooking process can change the dimensions of rice grains. The impact of set times of cooking or steaming process on the characteristics such as length, width, and height of two varieties of rice, namely, long-grain white and parboiled, was investigated. The measurements of the dimension characteristics obtained at different times of the cooking process were converted to functional data. Different methods of multivariate functional data analysis, namely, functional multivariate analysis of variance, functional discriminant coordinates, and cluster analysis, were applied to discover the differences between the two varieties and the two heat treatment methods.

## 1. Introduction

### 1.1. Background

The basis of a healthy diet is the consumption of a variety of products characterized by a high quality and low degree of processing, which ensures the supply of the body with necessary nutrients ([1], [2]). Rice is among the products increasingly used in a variety of meals. In the countries of the Far East, rice occupies a place in the hierarchy of food products which corresponds to that of bread in the European culture (Vecchia [3]).

**Figure 1:**
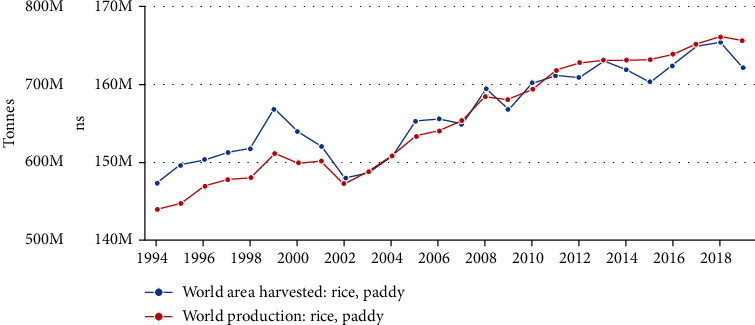
World production of rice in 1994-2019 (FAO).

Rice is the most widely produced grain on a global scale. Obtaining a few crops per year means that its popularity in countries with a suitable climate for its cultivation is constantly growing. The chart prepared by FAO shows the amount of world production in 1994-2019 presented in Figure 1. There is a continuous upward trend in both production and productivity per hectare. Over the past decade, world rice production increased by less than 15%. Rice production is mostly concentrated in Asia, where over 90% of its crops come from, and the unrivaled leader in production is China (http://www.fao.org/faostat/en/#data/QC/visualize).

An increase in the production of agricultural food products is always caused by an increase in the consumption of a given product. For rice, consumption has increased by around 17% over the last decade (2010–2020).

Cooking is the most important unit operation carried out for preparing food for consumption. It involves a combination of heat and mass transfer during which several components of the cooked material undergo phase changes ([4]). While cooking rice, the most important change that occurs is the sticking of starch due to elevated temperatures and increased humidity. Gelatinization of starch caused by the breaking of intermolecular hydrogen bonds increases its water absorption ([5]).

Magnetic resonance imaging enables the characterization of changes taking place in heat and moisture distribution while cooking rice grains in water. It has been shown that the generated three-dimensional images show the heat and mass distribution in grains cooked for 5-32 minutes indicating a more intensively progressive cooking process along cracks and micropores ([6], [7]). Detailed studies of temperature and water content distribution during cooking were performed in the rice kernels of *Oryza sativa* L. Japonica cv. Nipponbare using nuclear magnetic resonance. It was observed that the increase in water content from the start of cooking till 12 minutes was due to the absorption of free water by the grains, whereas the decrease in water content after 15 and 25 minutes was caused by the progressive gluing of starch ([8], [9]).

Research on the influence of cooking with excess water at different temperatures (80, 100, 120, and 140°C) and pressures (0, 0.1, 0.3, and 0.5 MPa) on the textural and morphological properties of aromatic Jasmine rice grains of the Thai variety KDML 105 showed full gelatinization of rice cooked at 80, 100, 120, and 140°C after 22, 14, 8, and 6 minutes of cooking, respectively. The increase in temperature and pressure during cooking was accompanied by an increase in softness and porosity due to the formation of a more spatially complex starch matrix in the inner layers of the grains. In contrast to temperature, a higher pressure reduced the porosity of the outer layers of the cooked grains ([10], [11]).

Depending on the species, rice is characterized by a diversified content of amylose (15-27%), which is related to the length of the grains. Long-grain cultivars have the highest amylose content, and a decrease in this content was noted along with decreasing grain length. High amylose content extends the cooking time and increases the degree of water absorption by the cooked raw material ([12], [13]). Rice grains containing a higher level of amylose show a more pronounced increase in size, greater cohesiveness, and hardness after cooking compared to low-amylose varieties ([14], [15]). Low-amylose level is associated with the soft structure of rice grains, and amylose degradation progresses with the cooking time ([16], [17]).

The quality of the cooked rice grains is determined by the degree of water absorption and the degree of change in volume. Both these parameters have a direct impact on the nutritional and taste properties that influence consumer acceptability ([18], [19]).

Modern methods applied for cooking rice mainly use boiling water, superheated steam, and microwaves. To achieve the appropriate degree of cooking, it is necessary to define the parameters based on which the optimally cooked grains will be identified. An analytical determinant of cooking in the case of rice is the degree of stickiness of the starch it contains. To achieve gluing, apart from the right temperature, an adequate amount of water is required, which will be absorbed by the swelling starch. The most intense water absorption by rice grains occurs in the initial cooking phase and slows down after 20 minutes of cooking ([20], [21]). It was reported that optimally cooked rice showed 90% sticky starch after 20 minutes of cooking with excess water ([22], [23], [24]). To obtain the best possible quality of cooked rice, it should be cooled immediately after cooking. Cooling the rice after cooking with excess water has been shown to improve the quality of the rice, but it greatly extended the technological operation time ([12], [25], [13]).

In the food industry of the Far East (China, Japan, and Thailand), there are automatic lines for cooking rice with a capacity of up to 600 kg/h (http://satake-group.com, http://www.diytrade.com). These lines use a system of containers in which a portion of rice is cooked in a properly set amount of water. Cooking is done by supplying the rice and water mixture with heat by gas or electric heating or under the influence of a microwave field (http://www.made-in-china.com, http://www.acesystem.co.jp). For high-tech rice cookers, heat is supplied using several methods in one unit (http://www.aiho.co.jp). For efficient operation of such devices, it is necessary to obtain data on the amount dosed from individual containers of rice and water ([26], http://www.daneng.com.sg). For economic reasons, the most frequently used method of rice cooking is cooking in excess of water. In this way, in a relatively short time we obtain a product with appropriate technological parameters; however, observing individual grains of rice cooked in such a way, we can see a multitude of damages and deformations caused by fast softening of grains exposed to water and temperature. The individual structure of the grains is destroyed and the obtained mass is characterized by very high digestibility. The treatment of rice grains with water vapor at atmospheric pressure is a less invasive method. In this method, the starch grains swell much more slowly so that the whole grain does not disintegrate ([27]; [26]; [28]). Defining the characteristics related to changes in the dimensions of grains of individual rice varieties is an important aspect for designing the assumptions of new devices for cooking rice grains in continuous systems, which will carry out the cooking process in the flow (http://www.daneng.com.sg, http://n.leying.cn) ([29]).

### 1.2. Findings

In this study, we consider two varieties of rice: long-grain white and parboiled. The grains of these varieties were steamed or boiled in water, and thus, we had four rice research groups. Grains in these groups were characterized by three parameters: length, width, and height. These features were measured before cooking and after 5, 10, 15, 20, 25, and 30 minutes of cooking, respectively, which resulted in time series data. These series were converted into continuous functions *x*(*t*) of time *t* on the interval [0, 30]. The processed data are called functional data ([30]).

The research question is as follows: can homogeneous subgroups be distinguished from among the four groups of rice grains described by functional data? In this work, we present a research methodology that allows us to answer this question. The discussed statistical methods are universal and can be used to study any groups of objects characterized by multidimensional functional data, and not only grains of different rice varieties. Currently, statistical methods for functional data are being developed because functional data have many useful properties.

There are numerous reasons to model a time series as a continuous function (elements of a certain functional space), one of which is that functional data have more advantages compared to other ways of time series representation. In particular, the multivariate functional discriminant coordinate analysis (MFDCA) derived in the present study has the following statistical advantages.

Firstly, functional data are normally used to cope with the problem of missing observations, which is inevitable in many areas of applied research. Unfortunately, most methods related to data analysis require a complete time series. Removing time series having missing observations from a data set is simply one of the popular solutions, but this can lead, and in most cases does lead, to serious data loss. Another possibility is to use one of the many methods available for missing data prediction, but in that case the results will depend on the interpolation method. Contrary to these approaches, in the case of functional data, the problem of missing observations is resolved by expressing time series in the form of a continuous function set.

Secondly, in the statistical development of MFDCA, the structure of observations is naturally maintained when using the functional data, i.e., the temporal link is maintained and the information regarding any measurement is taken into account. Consequently, the robustness of results is assumed.

Thirdly, the moments of observation do not have to be equally spaced in a particular time series, which can be a major advantage in online applications.

Fourthly, when using functional data, one can avoid the problem of dimensionality. When the total number of time points, in which the observations are made, exceeds the number of the examined time series data, most statistical methods do not provide satisfactory results due to misleading false estimates. However, this issue can be avoided in the case of functional data because the time series are replaced by a set of continuous representative functions that are independent of the time points in which the observation is made.

### 1.3. Results and Purpose

The statistical methodology consists of four steps. In the first step, we transform the original time series data into functional data. In the second step, we use the functional multivariate analysis of variance (FMANOVA), which verifies the hypothesis that there are no differences between the vectors of mean functions representing the four studied groups. After rejecting this hypothesis, in the next step, we construct functional discriminant coordinates that allow us to visualize how the relative position of the four studied groups looks like on the plane. In the last step, to confirm the existence of homogeneous subgroups, we use a cluster analysis based on the construction of a dendrite (minimum spanning tree) connecting the studied groups.

The main purpose of the paper is to find out differences and similarities between rice varieties after the cooking process using statistical methods of functional data analysis.

The paper is organized as follows.  Section 2 describes the material and presents the statistical methodology of the research.  Section 3 contains the results and discussion. Finally, Section 4 provides a summary of the studies performed.

## 2. Materials and Methods

### 2.1. Schematic Overview of the Experimental Program

**Figure 2:**
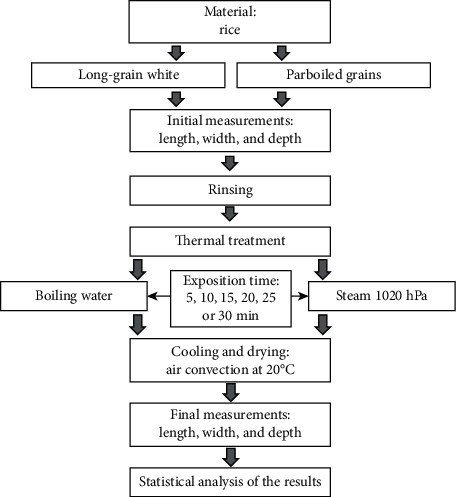
Block diagram of the conducted research.

The research was carried out in accordance with the developed scheme of investigation presented in Figure 2. Two types of rice, i.e., parboiled and long-grain white, were used in the study. The selection criteria applied for the study material were as follows: high quality, high degree of size and color uniformity, and the lowest percentage of impurities. The parboiled rice was produced by Müller's Mühle GmbH, and the long-grain white rice was purchased from Sawexs. The materials used in the study were qualified as class I products. No signs of infestation with microorganisms or pests were detected in the rice, and it was not pretreated to shorten the cooking time.

### 2.2. Sample Collection and Preparation

Samples of 10 grains each were collected from the study materials to characterize the changes in the kernel size caused by the progressive cooking process in various conditions. The extreme dimensions of the grains were measured, and then the rice was boiled for 5, 10, 15, 20, 25, or 30 minutes in excess water or in a water vapor atmosphere at atmospheric pressure (approximately 1020 hPa). After cooking, the grains were cooled in an air stream at 15°C, and the main dimensions were measured again. The size parameters were measured using an Olympus BX61 automatic microscope coupled with a PC, a DP12 camera, and Olympus DP Soft software (all the detailed parameters of the equipment are available on the manufacturer's website https://www.olympus-lifescience.com). The use of optical software-supported measurement methods allowed precise noncontact measurement of the elements of high viscosity and delicate consistency. The distances between the extreme points on a rice grain or its cross-section were determined with an accuracy of 0.001 mm with the use of the following Olympus UIS2-Series Biological Objective lenses: “10x U Plan FL N-0.30 NA” and “20x U Plan FL N-0.50 NA.” The extreme dimensions were determined by the image analysis program “DP Soft.”

### 2.3. Statistical Methodology

#### 2.3.1. Functional Data

We assume that the object being classified is described by a *p*-dimensional random process **X** = (*X*_1_, ⋯,*X*_*p*_)′ ∈ *L*_2_^*p*^(*I*), where *L*_2_(*I*) is the Hilbert space of square-integrable functions, **A**′ denotes a transposition of **A**, and *I* is an interval [0, *T*], *T* > 0. The Hilbert space *L*_2_^*p*^(*I*) is equipped with the following inner product:

(1) $$\begin{array}{c} \langle u,v\rangle =\int_{I}u^{\prime }\left(t\right)v\left(t\right)dt. \end{array}$$

Moreover, we assume that the *k*th component of vector **X** can be represented by a finite number of orthonormal basis functions {*φ*_*b*_}:

(2) $$\begin{array}{c} X_{k}\left(t\right)=\overset{B_{k}}{\underset{b=0}{\sum }}\alpha_{kb}\varphi_{b}\left(t\right),\;\;t\in I,\;k=1,\cdots ,p, \end{array}$$

where *α*_*k*0_, *α*_*k*1_, ⋯, *α*_*kB*_*k*__ are the unknown coefficients.

Let *α* = (*α*_10_, ⋯,*α*_1*B*_1__, ⋯,*α*_*p*0_, ⋯,*α*_*pB*_*p*__)′ and

(3) $$\begin{array}{c} \Phi \left(t\right)=\left(\begin{array}{cccc} \varphi \prime_{1}\left(t\right) & 0^{\prime } & \cdots & 0^{\prime }\\ 0^{\prime } & \varphi \prime_{2}\left(t\right) & \cdots & 0^{\prime }\\ \cdots & \cdots & \cdots & \cdots \\ 0^{\prime } & 0^{\prime } & \cdots & \varphi \prime_{p}\left(t\right) \end{array}\right), \end{array}$$

where *φ*_*k*_(*t*) = (*φ*_0_(*t*), ⋯,*φ*_*B*_*k*__(*t*))′, *k* = 1, ⋯, *p*.

Using the above matrix notation, process **X** can be represented as follows:

(4) $$\begin{array}{c} X\left(t\right)=\Phi \left(t\right)\alpha . \end{array}$$

This indicates that the realizations of process **X** are in a finite dimensional subspace of *L*_2_^*p*^(*I*).

We can estimate the vector *α* from *n* independent realizations *x*_1_, ⋯, *x*_*n*_ of the random process **X** (functional data), and we denote this estimator by **a**.

Typically, data are recorded at discrete moments in time. Let *x*_*kj*_ denote an observed value of the feature *X*_*k*_, *k* = 1, ⋯, *p* at the *j*th time point *t*_*j*_, where *j* = 1, ⋯, *J*. Then, our data consist of the *pJ* pairs (*t*_*j*_, *x*_*kj*_). These discrete data can be smoothed by continuous functions *x*_*k*_, and *I* is a compact set such that *t*_*j*_ ∈ *I*, for *j* = 1, ⋯, *J*.

The process of transformation of discrete data into functional data is described by Ramsay and Silverman [30] and Górecki et al. [31].

#### 2.3.2. Functional Multivariate Analysis of Variance

In the MANOVA for functional data, we test the equality of vectors of mean functions in *L* populations. Let **X**_*ij*_ = (*X*_*ij*1_, ⋯,*X*_*ijp*_)′ ∈ *L*_2_^*p*^(*I*), *i* = 1, ⋯, *L*, *j* = 1, ⋯, *n*_*i*_ be *L* independent samples from these populations consisting of *p*-dimensional stochastic processes with mean vectors **μ**_*i*_. Let *n* = *n*_1_ + ⋯+*n*_*L*_ be the total sample size. Thus, in FMANOVA, we verify the following null hypothesis:

(5) $$\begin{array}{c} H_{0}:\mu_{1}\left(t\right)=\cdots =\mu_{L}\left(t\right),\;\;t\in I, \end{array}$$

against the alternative hypothesis *H*_1_, which is its negation. For this statistical hypothesis testing problem, we will use the permutation tests based on a basis function representation and the tests based on random projections proposed by Górecki and Smaga [32, 33]. They are implemented in the R package fdANOVA ([33]).

The permutation tests based on a basis function representation use the representation of the **X**_*ij*_ components as introduced in Section 2.3.1, i.e., we have

(6) $$\begin{array}{c} X_{ij}\left(t\right)=\left(\begin{array}{c} \alpha_{ij1}\\ \vdots \\ \alpha_{ijp} \end{array}\right)\varphi \left(t\right)=\alpha_{ij}\varphi \left(t\right), \end{array}$$

where *α*_*ijm*_ = (*α*_*ijm*1_, ⋯, *α*_*ijmB*_*m*__, 0, ⋯, 0) ∈ ℝ^*BM*^, *φ*(*t*) = (*φ*_1_(*t*), ⋯,*φ*_*BM*_(*t*))′, *t* ∈ *I* and *i* = 1, ⋯, *L*, *j* = 1, ⋯, *n*_*i*_, *m* = 1, ⋯, *p*, *BM* = max{*B*_1_, ⋯, *B*_*p*_}.

Let

(7) $$\begin{array}{c} \bar{X}\left(t\right)=\frac{1}{n}\overset{L}{\underset{i=1}{\sum }}\overset{n_{i}}{\underset{j=1}{\sum }}X_{ij}\left(t\right),\\ {\bar{X}}_{i}\left(t\right)=\frac{1}{n_{i}}\overset{n_{i}}{\underset{j=1}{\sum }}X_{ij}\left(t\right), \end{array}$$

where *i* = 1, ⋯, *L* and *t* ∈ *I* be the total sample mean function and the group sample mean functions, respectively. Then, similarly to MANOVA ([34]), the following matrices were used to construct test statistics for (5):

(8) $$\begin{array}{c} E=\overset{L}{\underset{i=1}{\sum }}\overset{n_{i}}{\underset{j=1}{\sum }}\int_{I}\left(X_{ij}\left(t\right)-{\bar{X}}_{i}\left(t\right)\right){\left(X_{ij}\left(t\right)-{\bar{X}}_{i}\left(t\right)\right)}^{\prime }dt,\\ H=\overset{L}{\underset{i=1}{\sum }}n_{i}\int_{I}\left({\bar{X}}_{i}\left(t\right)-\bar{X}\left(t\right)\right){\left({\bar{X}}_{i}\left(t\right)-\bar{X}\left(t\right)\right)}^{\prime }dt. \end{array}$$

Using (6), we obtain **E** = **C**_1_ − **C**_2_ and **H** = **C**_2_ − **C**_3_, where

(9) $$\begin{array}{c} C_{1}=\overset{L}{\underset{i=1}{\sum }}\overset{n_{i}}{\underset{j=1}{\sum }}\alpha_{ij}{\alpha_{ij}}^{\prime },\\ C_{2}=\overset{L}{\underset{i=1}{\sum }}\frac{1}{n_{i}}\overset{n_{i}}{\underset{j=1}{\sum }}\overset{n_{i}}{\underset{m=1}{\sum }}\alpha_{ij}{\alpha_{im}}^{\prime },\\ C_{3}=\frac{1}{n}\overset{L}{\underset{i=1}{\sum }}\overset{n_{i}}{\underset{j=1}{\sum }}\overset{L}{\underset{v=1}{\sum }}\overset{n_{v}}{\underset{u=1}{\sum }}\alpha_{ij}{\alpha_{vu}}^{\prime }. \end{array}$$

Thus, the matrices **E** and **H** are expressed by the coefficients of basis representation only. The following test statistics for (5) are constructed based on those appearing in the classical MANOVA:

1. the Wilks lambda *W* = det(**E**)/det(**E** + **H**)
2. the Lawley-Hotelling trace LH = trace(**HE**^−1^)
3. the Pillai trace *P* = trace(**H**(**H** + **E**)^−1^)
4. the Roy maximum root *R* = *λ*_max_(**HE**^−1^)

where *λ*_max_(**M**) is the maximum eigenvalue of a matrix **M**. To construct tests based on these test statistics, we use the permutation method. Hence, we have four permutation tests according to a basis function representation. The Fourier basis and 1000 permutation samples were used in the computation.

Now, let us focus on the tests based on random projections. Assume that *ξ* is a Gaussian distribution on *L*_2_(*I*), and each of its one-dimensional projections is nondegenerate. Suppose that *μ*_*ij*_ ∈ *L*_2_(*I*), where *μ*_*ij*_ are the following components: *μ*_*i*_, *i* = 1, ⋯, *L*, *j* = 1, ⋯, *p*. If null hypothesis (5) is true, then for every **h** = (*h*_1_, ⋯,*h*_*p*_)′ ∈ *L*_2_^*p*^(*I*), this holds:

(10) $$\begin{array}{c} H_{0}^{h}:{\left(\langle \mu_{11},h_{1}\rangle ,\cdots ,\langle \mu_{1p},h_{p}\rangle \right)}^{\prime }=\cdots ={\left(\langle \mu_{L1},h_{1}\rangle ,\cdots ,\langle \mu_{Lp},h_{p}\rangle \right)}^{\prime }. \end{array}$$

On the other hand, when *H*_0_ is not true, for (*ξ*×⋯×*ξ*)—almost every **h** ∈ *L*_2_^*p*^(*I*), *H*_0_^**h**^ fails. Thus, a test for the MANOVA problem can be applied to analyze the FMANOVA problem by using the following testing procedure:

1. Choose the number of projections *k* ∈ *ℕ*
2. Choose, with Gaussian distribution, the functions *h*_*mr*_ ∈ *L*_2_(*I*), *m* = 1, ⋯, *p*, *r* = 1, ⋯, *k*
3. Compute the projections

(11) $$\begin{array}{c} P_{ijm}^{r}=\frac{\int_{I}X_{ijm}\left(t\right)h_{mr}\left(t\right)dt}{{\left(\int_{I}h_{mr}^{2}\left(t\right)dt\right)}^{1/2}}, \end{array}$$

for *i* = 1, ⋯, *L*, *j* = 1, ⋯, *n*_*i*_, *m* = 1, ⋯, *p*, *r* = 1, ⋯, *k*.

(4) For each *r* ∈ {1, ⋯, *k*}, apply the chosen MANOVA test for vectors **P**_*ij*_^*r*^ = (*P*_*ij*1_^*r*^, ⋯,*P*_*ijp*_^*r*^)′, *i* = 1, ⋯, *L*, *j* = 1, ⋯, *n*_*i*_. Let *p*_1_, ⋯, *p*_*k*_ denote the resulting *p* values
(5) Compute the following final *p* value for (5)

(12) $$\begin{array}{c} \inf\left\{\frac{kp_{\left(r\right)}}{r},r=1,\cdots ,k\right\}, \end{array}$$

where *p*_(1)_ ≤ ⋯≤*p*_(*k*)_ are the ordered *p* values obtained in step 4.

For step 4, we use the abovementioned MANOVA tests, i.e., the Wilks lambda test, the Lawley-Hotelling trace test, the Pillai trace test, and the Roy maximum root test. We denote them as Wp, LHp, Pp, and Rp tests, respectively. We also consider their permutation versions as well as different Gaussian distributions *ξ*, i.e., the Gaussian white noise and the Brownian motion. The number of random projections was set to *k* = 30, as suggested in the literature [32, 35].

#### 2.3.3. Functional Discriminant Coordinate Analysis

Let us consider the case where the samples originate from *L* groups. We would often like to present them graphically, to see their configuration, or to eliminate outlying observations. However, it may be difficult to produce such a presentation even if only three features are observed. The space of discriminant coordinates is convenient for using various classification methods (methods of discriminant analysis).

Our purpose is to construct a discriminant coordinate based on multivariate functional data:

(13) $$\begin{array}{c} U=\langle u,X\rangle , \end{array}$$

such that their between-class variance is maximal compared with the total variance, where **u** ∈ *L*_2_^*p*^(*I*). More precisely, the first functional discriminant coordinate *U*_1_ = 〈**u**_1_, **X**〉 is defined as follows:

(14) $$\begin{array}{c} \lambda_{1}=\underset{u\in L_{2}^{p}\left(I\right)}{\sup}\frac{{\text{Var}}_{B}\left(\langle u,X\rangle \right)}{{\text{Var}}_{T}\left(\langle u,X\rangle \right)}=\frac{{\text{Var}}_{B}\left(\langle u_{1},X\rangle \right)}{{\text{Var}}_{T}\left(\langle u_{1},X\rangle \right)}, \end{array}$$

subject to the following constraint:

(15) $$\begin{array}{c} {\text{Var}}_{T}\left(\langle u_{1},X\rangle \right)=1, \end{array}$$

where Var_*B*_(〈**u**, **X**〉) and Var_*T*_(〈**u**, **X**〉) are, respectively, the between-class variance and total variance of discriminant coordinate *U*_1_. Condition (15) ensures the uniqueness of the first discriminant coordinate *U*_1_.

Now, let us assume that the process **X** and the vector weight function **u** can be represented as follows:

(16) $$\begin{array}{c} X\left(t\right)=\Phi \left(t\right)\alpha ,\\ u\left(t\right)=\Phi \left(t\right)\gamma , \end{array}$$

where *t* ∈ *I*, *α*, *γ* ∈ ℝ^*K*+*p*^, and *K* = *B*_1_ + ⋯+*B*_*p*_.

Hence, the between-class variance of the inner product 〈**u**, **X**〉 is as follows:

(17) $$\begin{array}{c} {\text{Var}}_{B}\left(\langle u,X\rangle \right)=\gamma^{\prime }{\text{Var}}_{B}\left(\alpha \right)\gamma , \end{array}$$

and the total variance is as follows:

(18) $$\begin{array}{c} {\text{Var}}_{T}\left(\langle u,X\rangle \right)=\gamma^{\prime }{\text{Var}}_{T}\left(\alpha \right)\gamma , \end{array}$$

where Var_*B*_(*α*) = **B** and Var_*T*_(*α*) = **T** are, respectively, the matrices of the sum of squares and products of between-class variance and total variance. For the first functional discriminant coordinate of the process **X**, we have

(19) $$\begin{array}{c} \lambda_{1}=\underset{u\in L_{2}^{p}\left(I\right)}{\sup}\frac{{\text{Var}}_{B}\left(\langle u,X\rangle \right)}{{\text{Var}}_{T}\left(\langle u,X\rangle \right)}=\underset{\gamma \in {\mathbb{R}}^{K+p}}{\sup}\frac{\gamma^{\prime }{\text{Var}}_{B}\left(\alpha \right)\gamma }{\gamma^{\prime }{\text{Var}}_{T}\left(\alpha \right)\gamma }=\frac{{\gamma_{1}}^{\prime }B\gamma_{1}}{{\gamma_{1}}^{\prime }T\gamma_{1}}, \end{array}$$

where

(20) $$\begin{array}{c} \gamma^{\prime }{\text{Var}}_{T}\left(\alpha \right)\gamma =1. \end{array}$$

Similarly, we can construct the *k*th functional discriminant coordinate:

(21) $$\begin{array}{c} U_{k}=\langle u_{k},X\rangle , \end{array}$$

where the vector weight function **u**_*k*_ is defined as follows:

(22) $$\begin{array}{c} \lambda_{k}=\underset{u\in L_{2}^{p}\left(I\right)}{\sup}\frac{{\text{Var}}_{B}\left(\langle u,X\rangle \right)}{{\text{Var}}_{T}\left(\langle u,X\rangle \right)}=\underset{\gamma \in {\mathbb{R}}^{K+p}}{\sup}\frac{\gamma^{\prime }{\text{Var}}_{B}\left(\alpha \right)\gamma }{\gamma^{\prime }{\text{Var}}_{T}\left(\alpha \right)\gamma }=\frac{{\gamma_{k}}^{\prime }B\gamma_{k}}{{\gamma_{k}}^{\prime }T\gamma_{k}}, \end{array}$$

subject to the following constraint:

(23) $$\begin{array}{c} {\text{Cov}}_{T}\left(\gamma_{\kappa_{1}}^{\prime }T\gamma_{\kappa_{1}},\gamma_{\kappa_{2}}^{\prime }T\gamma_{\kappa_{2}}\right)=\left\{\begin{array}{cc} 1, & \kappa_{1}=\kappa_{2},\\ 0, & \kappa_{1}\neq \kappa_{2}, \end{array}\right. \end{array}$$

where *κ*_1_, *κ*_2_ = 1, ⋯, *s*, *s* = min(*K* + *p*, *L* − 1). The expression (*λ*_*k*_, **u**_*k*_) will be called the *k*th discriminant system of the process **X**.

Let us now define the classical discriminant coordinates for the random vector *α*. The *k*th discriminant component of this vector satisfies

(24) $$\begin{array}{c} \lambda_{k}^{*}=\underset{\gamma \in {\mathbb{R}}^{K+p}}{\sup}{\text{Var}}_{B}\left(\langle \gamma ,\alpha \rangle \right)={\text{Var}}_{B}\left(\langle \gamma_{k},\alpha \rangle \right)={\gamma_{k}}^{\prime }{\text{Var}}_{B}\left(\alpha \right)\gamma_{k}={\gamma_{k}}^{\prime }B\gamma_{k}, \end{array}$$

subject to the following constraint:

(25) $$\begin{array}{c} {\text{Cov}}_{T}\left(\gamma_{\kappa_{1}}^{\prime }T\gamma_{\kappa_{1}},\gamma_{\kappa_{2}}^{\prime }T\gamma_{\kappa_{2}}\right)=\left\{\begin{array}{cc} 1, & \kappa_{1}=\kappa_{2},\\ 0, & \kappa_{1}\neq \kappa_{2}. \end{array}\right. \end{array}$$

The pair (*λ*_*k*_^∗^, *γ*_*k*_) will be called the *k*th discriminant system of the random vector *α*.

We may extend these considerations to the second discriminant system and so on. Thus, the following theorem is true.

**Theorem 1.**

The *k*th discriminant system (*λ*_*k*_, **u**_*k*_) of the stochastic process **X** is related to the *k*th discriminant system (*λ*_*k*_^∗^, *γ*_*k*_) of the random vector *α* by

(26) $$\begin{array}{c} \lambda_{k}=\lambda_{k}^{*},\\ u_{k}\left(t\right)=\Phi \left(t\right)\gamma_{k},\\ \;\;t\in I, \end{array}$$

where *k* = 1, ⋯, *s*, *s* = min(*K* + *p*, *L* − 1) = rank**B**.

At a given time point *t*, the greater the absolute value of a component of the vector weight function, the greater the contribution in the structure of the given functional discriminant coordinate, from the process **X** corresponding to that component. The total contribution of a particular original process *X*_*i*_(*t*) in the structure of a particular functional discriminant coordinate is equal to the area under the module weighting function corresponding to this process.

In practice, the matrices Var_*B*_(*α*) and Var_*T*_(*α*) are unknown and must be estimated based on the sample. Let **x**_*i*1_, ⋯, **x**_*in*_*i*__ be a sample belonging to the *i*th class, where *i* = 1, ⋯, *L*. The function **x**_*ij*_ has the following form:

(27) $$\begin{array}{c} x_{ij}\left(t\right)=\Phi \left(t\right)a_{ij}. \end{array}$$

Let

(28) $$\begin{array}{c} \bar{a}=\frac{1}{n}\overset{L}{\underset{i=1}{\sum }}\overset{n_{i}}{\underset{j=1}{\sum }}a_{ij},\\ {\bar{a}}_{i}=\frac{1}{n_{i}}\overset{n_{i}}{\underset{j=1}{\sum }}a_{ij},\\ i=1,\cdots ,L. \end{array}$$

Then, we have

(29) $$\begin{array}{c} \hat{B}=\frac{1}{L-1}\overset{L}{\underset{i=1}{\sum }}n_{i}\left({\bar{a}}_{i}-\bar{a}\right){\left({\bar{a}}_{i}-\bar{a}\right)}^{\prime },\\ \hat{T}=\frac{1}{n-1}\overset{L}{\underset{i=1}{\sum }}\overset{n_{i}}{\underset{j=1}{\sum }}\left(a_{ij}-\bar{a}\right){\left(a_{ij}-\bar{a}\right)}^{\prime }, \end{array}$$

where *n* = ∑_*i*=1_^*L*^*n*_*i*_.

Next, we find the nonzero eigenvalues ${\hat{\lambda }}_{1}^{*}\geq {\hat{\lambda }}_{2}^{*}\geq \cdots \geq {\hat{\lambda }}_{s}^{*}$ and the corresponding eigenvectors ${\hat{\gamma }}_{1},{\hat{\gamma }}_{2},\cdots ,{\hat{\gamma }}_{s}$ of the matrix ${\overset{\wedge }{T}}^{-1}\hat{B}$, where *s* = min(*K* + *p*, *L* − 1). Furthermore, the *k*th discriminant system of the random process **X** has the following form:

(30) $$\begin{array}{c} {\hat{\lambda }}_{k}={\hat{\lambda }}_{k}^{*},\\ {\hat{u}}_{k}\left(t\right)=\Phi \left(t\right){\hat{\gamma }}_{k},\\ k=1,\cdots ,s. \end{array}$$

Hence, the coefficients of the projection of the *j*th realization **x**_*ij*_ of the process **X** belonging to the *i*th class on the *k*th functional discriminant coordinate are equal to

(31) $$\begin{array}{c} {\hat{U}}_{ijk}=\langle {\hat{u}}_{k},x_{ij}\rangle =\hat{\gamma }\prime_{k}a_{ij}, \end{array}$$

for *j* = 1, ⋯, *n*_*i*_, *k* = 1, ⋯, *s*, *i* = 1, ⋯, *L*.

The plots of the pairs $\left({\hat{U}}_{ij1},{\hat{U}}_{ij2}\right)$ allow visualizing the relative position of groups in the two-dimensional space. Since the configuration obtained is deemed to be optimum in terms of discrimination among groups, wide overlaps should be considered as a sign of either no or small differences between the groups involved.

#### 2.3.4. Cluster Analysis Based on Minimum Spanning Tree

Here, we briefly discuss the cluster analysis method based on minimum spanning tree. According to the graph theory, a dendrite is synonymous with a tree. A dendrite or tree is a graph (every two vertices are connected by a path) that does not contain cycles. A minimum dendrite is the one in which the sum of the weights at the edges is minimal. The most common weight is distances. The construction of dendrite was described by a group of Wrocław mathematicians ([36]). Two extremely simple procedures are used to construct a minimal dendrite. The first is the Kruskal algorithm:

1. Select the edge with the shortest length
2. From the remaining edges, choose the one with the shortest length which does not lead to the cycle (choose any connection of equal length)
3. Repeat the previous step to complete the construction of the minimal dendrite

The second is the Prim algorithm which starts with the tree consisting of one vertex, constantly adding the shortest edge of the tree.

The shortest dendrite can be used in cluster analysis. The idea behind this approach is to remove from the minimum dendrite all edges, the length of which is greater than the common critical value *d*. We calculate the mean value $\bar{x}$ and the standard deviation *s* from the length of all edges of the shortest dendrite and assume $d=\bar{x}+s$. The vertices that remain connected in a minimal dendrite form a cluster.

## 3. Research and Discussion

In this study, we consider four groups of rice grains:

1. Long-grain white (steaming)
2. Long-grain white (boiling)
3. Parboiled (steaming)
4. Parboiled (boiling)

In the first step, the original data, which were recorded in the form of a time series, were transformed into continuous functions (using the method described by Ramsay and Silverman [30] and Górecki et al. [31]). We consider these functions on the interval [0, *T*] = [0, 30] because the seven time points (before cooking and after 5, 10, 15, 20, 25, and 30 minutes of cooking), in which the values of three features of the rice grains were observed, i.e., the length, width, and height, lie within this range. This transformation uses the first five Fourier base functions of the form

(32) $$\begin{array}{c} \phi_{0}\left(t\right)=\frac{1}{\sqrt{T}},\;\\ \phi_{2k-1}\left(t\right)=\sqrt{\frac{2}{T}}\sin\left(\frac{2\pi kt}{T}\right),\\ \phi_{2k}\left(t\right)=\sqrt{\frac{2}{T}}\cos\left(\frac{2\pi kt}{T}\right), \end{array}$$

where *t* ∈ [0, *T*], *k* = 1, 2, ⋯.

**Figure 3:**
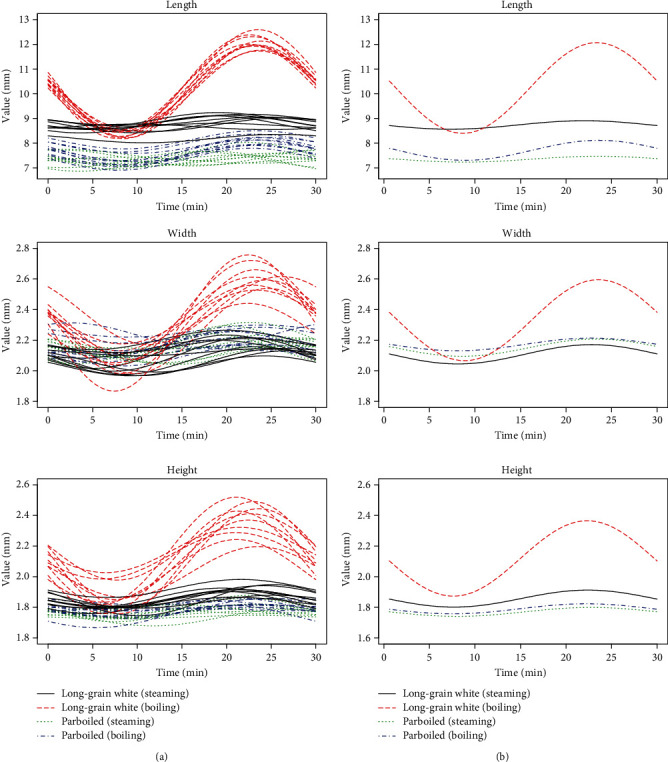
Trajectories of functional data (a) and sample mean functions (b) shown separately for each variable.

Figure 3 shows the continuous functions (functional data) obtained for the four groups of rice with marked average functions in each group. Figure 3 also shows that the biggest difference of characteristics of compared groups are observed after 20-25 minutes of thermal treatment, but the smallest differences are observed after 5-10 minutes.

In the second step, we tested the general null hypothesis (5) regarding the equality of the mean function vectors in the four groups of rice. The sample mean functions for each variable are presented in Figure 3. For this purpose, we applied all the FMANOVA tests described in Section 2.3.2. The *p* values of all permutation tests were equal to zero, and those of the standard projection tests (i.e., the Wp, LHp, Pp, and Rp tests with the Gaussian white noise and the Brownian motion) were close to zero, i.e., the largest *p* value was equal to 1.286826 · 10^−13^. Thus, all the FMANOVA tests rejected the null hypothesis (5), even at a very low significance level. This indicates that the four groups of rice are significantly different in mean functions.

**Figure 4:**
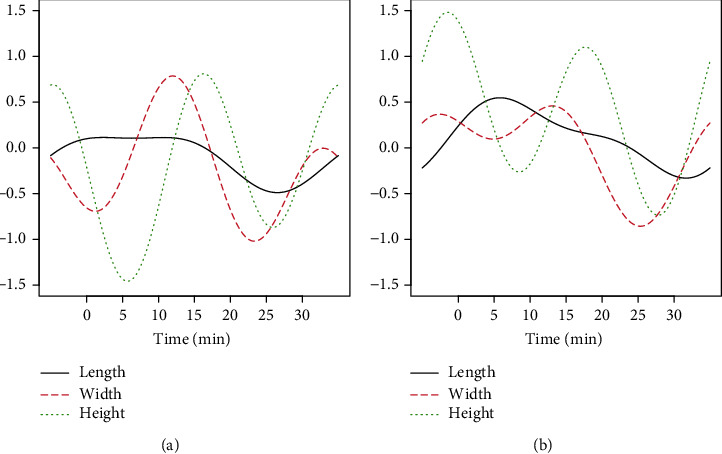
Weight functions for the first (a) and second (b) functional discriminant coordinates.

In the third step, three functional discriminant coordinates were constructed for the four distinguished groups of rice grains. According to the theory of construction of these coordinates, their number is equal to *s* = min(*K* + *p*, *L* − 1), where *K* = *B*_1_ + ⋯+*B*_*p*_, *p* is the number of observed variables, and *L* is the number of distinguished groups. In our case, *K* = 12, *p* = 3, *L* = 4, and hence, *s* = min(15, 3) = 3. Thus, we obtained a three-dimensional space of functional discriminant coordinates. Each of the three coordinates has a different differentiating power (variance) for the four studied groups. The successive coordinates each has a differentiating power (expressed as a percentage) of 92.0, 7.3, and 0.7, respectively. Accordingly, the most significant is the first coordinate representing 92% of the overall variation, and the first two coordinates together represent 99.3%. The third coordinate is insignificant because its discriminant power is below 1%. The weight functions corresponding to the numerical coefficients in classical discriminant coordinates for the first two functional discriminant coordinates are shown in Figure 4.

The contribution made by the three observed variables to the structure of the first two discriminant coordinates (expressed as a percentage) is shown in Table 1. These represent the values of the fields under the weight function modules. In classical discriminant coordinates, the modules of numerical coefficients occurring in linear combinations representing the coordinates are taken into account. It can be noted that in the construction of both the first and the second discriminant coordinate, the greatest contribution is accounted for the height of the rice grains (65.3% and 50.6%, respectively).

**Figure 5:**
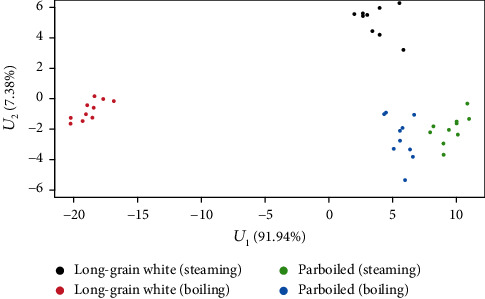
Plotted values of the first two functional discriminant coordinates.

The relative position of 40 rice grains, representing the four groups, in the system of the first two discriminant coordinates is shown in Figure 5.

As already mentioned, the differentiating power of these two coordinates is 99.3%. In Figure 5, one can see that the second group (long-grain white rice boiled in water) is very far from the other three groups. It proves that boiling of long-grain white rice in water has a different impact on the dimensions when we compare the dimensions of long-white rice after steaming or parboiled rice both after cooking or steaming.

Finally, we performed a cluster analysis based on a minimum spanning tree. In our calculations, we used the Kruskal algorithm and the Mahalanobis distance to construct a dendrite in a three-dimensional discriminant coordinate space. The vectors of the mean values of the four groups are shown in Table 2. The Mahalanobis distances between the groups are shown in Table 3. The connections in the dendrite are shown in Table 4.

Then, the mean lengths $\bar{x}$ of the dendrite edges, the standard deviations of these lengths *s*, and the critical value $d=\bar{x}+s=12.04+9.78=21.82$ were calculated.

**Figure 6:**
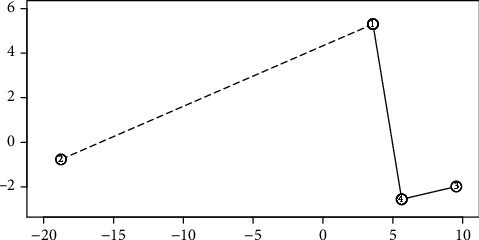
Minimum spanning tree.

In the system of the first two functional discriminant coordinates, a dendrite was spread on the means representing the four rice groups, and its division was made by marking edges longer than the critical value *d* with a dotted line (Figure 6).

It can be seen from the figure that two clusters are distinguished: a one-element cluster composed of group 2 and a three-element cluster composed of groups 1, 3, and 4. The Mahalanobis distance between groups 1 and 2 is 24.52 which is four times greater than the greatest distance between groups 1 and 4 belonging to the second cluster. Groups 1, 3, and 4 belonging to one cluster are homogeneous in the length, width, and height of rice grains (Table 5).

## 4. Conclusion

The paper describes the influence of the changes in the grain size of parboiled rice and long-grain white rice boiled in water and steamed at set processing times. The experimental results obtained for the stochastic process were converted to multidimensional functional data. The applied FMANOVA allowed rejecting the null hypothesis that the vectors of functional means in the compared groups do not differ significantly, at a significance level of 0.05. This confirmed the hypothesis that the studied rice groups had significant differences in the dimensions of grains. On the other hand, MFDCA showed that the experimental points presented in the system of the first two discriminant coordinates (explaining over 99% of the variability) formed four groups, which confirmed that the examined dimensions of rice grains differentiated the groups well. Additionally, the applied cluster analysis indicated that the dimensions of the long-grain white rice seeds boiled in water differ significantly from those of steamed long-grain white rice and both boiled and steamed parboiled rice. These conclusions from the cluster analysis will allow introducing additional design assumptions for new technological lines of rice cooking with periodic or continuous operation. They also indicate the possibility of using two methods for cooking rice grains in individual technological lines without the need for far-reaching modernization or lowering the assumed efficiency. The significant differences in the size changes demonstrated by the conducted analyses result from the modification of parboiled rice grains during the production process, when soaking and steaming under high pressure modify the components (mainly starch) of the endosperm. The dimensional changes caused by parboiling and steaming of rice grains during cooking are much smaller than those caused by immersion cooking of the grains of white varieties, where the structure loosens to a much greater extent than in other variants of the experiment.

## Figures and Tables

**(a):** 

First functional discriminant coordinate							
Variable	${\hat{\gamma }}_{10}$	${\hat{\gamma }}_{11}$	${\hat{\gamma }}_{12}$	${\hat{\gamma }}_{13}$	${\hat{\gamma }}_{14}$	Area	%
Length	-0.6172	1.1348	0.6911	-0.3726	0.1935	0.7658	6.6
Width	-1.3851	1.9187	0.0526	-1.8200	-1.9988	3.2306	28.1
Height	-1.3403	-0.8712	-1.0178	-4.1253	1.0350	7.5177	65.3

**(b):** 

Second functional discriminant coordinate							
Variable	${\hat{\gamma }}_{10}$	${\hat{\gamma }}_{11}$	${\hat{\gamma }}_{12}$	${\hat{\gamma }}_{13}$	${\hat{\gamma }}_{14}$	Area	%
Length	0.7644	1.6310	0.2588	0.5464	0.2715	3.2158	37.1
Width	-0.1435	1.6954	1.3107	-1.5916	0.0847	1.0715	12.3
Height	2.4974	0.7657	1.1232	-2.2123	3.3033	4.3897	50.6

**Table 2:** Mean values of the four groups.

Group number	Group	DC 1	DC 2	DC 3
1	Long-grain white (steaming)	-69.82	66.33	12.08
2	Long-grain white (boiling)	-92.16	60.26	12.44
3	Parboiled (steaming)	-63.85	59.04	13.52
4	Parboiled (boiling)	-67.76	58.47	10.86

**Table 3:** Mahalanobis distances.

Group number	1	2	3	4
1	0.00	23.15	9.53	8.22
2	23.15	0.00	28.36	24.52
3	9.53	28.36	0.00	4.76
4	8.22	24.52	4.76	0.00

**Table 4:** Connections in the dendrite.

Step	From group	To group	Distance
1	3	4	4.76
2	1	4	8.22
3	1	2	23.15

**Table 5:** Clusters.

Group number	Group	Cluster
1	Long-grain white (steaming)	1
2	Long-grain white (boiling)	2
3	Parboiled (steaming)	1
4	Parboiled (boiling)	1

## Data Availability

The data set is available from the third author upon request.
